# Sexual and reproductive healthcare needs of Iranian men: A cross-sectional study

**DOI:** 10.18502/ijrm.v16i12.3681

**Published:** 2018-01-28

**Authors:** Mojgan Javadnoori, Marjan Hajizadeh, Nahid Javadifar, Mohammad Hossein Haghighizadeh

**Affiliations:** ^1^Reproductive Health Promotion Research Center, Department of Midwifery, Faculty of Nursing and Midwifery, Ahvaz Jundishapur University of Medical Sciences, Ahvaz, Iran.; ^2^Bushehr University of Medical Sciences, Bushehr, Iran.; ^3^Department of Biostatistics and Epidemiology, School of Health, Ahvaz Jundishapur University of Medical Sciences, Ahvaz, Iran.

**Keywords:** *Reproductive health*, * Needs assessment*, * Men.*

## Abstract

**Background:**

The sexual and reproductive health (SRH) needs of men have received little attention in Iran's healthcare system. Developing appropriate strategies to meet men's needs requires careful assessment and recognition of their health needs.

**Objective:**

The objective of this study is to assess men's SRH needs and satisfaction with received services.

**Materials and Methods:**

This descriptive cross-sectional study was conducted with 1068 adult men aged between 20 and 60 years in Ahvaz in 2014. For obtaining the SRH services needs of men, in addition to the self-reported felt needs, expressed needs and unmet needs, a need assessment was also done using a questionnaire that was developed for the research; its validity and reliability were assessed.

**Results:**

The men's perceived, expressed and unmet needs for SRH services were, priority-wise, screening and diagnosis of male genital cancers (63.3%), receiving contraceptive methods (36%), diagnosis, and treatment of male sexual dysfunction (86.9%), respectively. Preventing sexually transmitted disease/AIDS (72.1%), using contraceptives correctly (39.5%), and resisting peer pressure (86.6%) were, respectively, the first felt, expressed, and unmet skills men needed. The results of multivariate logistical regression showed that there was a significant statistical correlation between men's SRH needs and their socio-demographic factors (age, marital/educational status, income) (p<0.05).

**Conclusion:**

Iranian men have many unmet SRH needs. Felt and expressed SRH needs were different. Educational and counseling services are as important as clinical services.

## 1. Introduction

One of the most important factors influencing health is individuals' access to effective, affordable, and culturally compatible healthcare services with the aim of reducing healthcare inequalities (1, 2). Failure to the provision of equal access to healthcare needs can lead to unmet healthcare needs (3). Unmet needs come from perceived needs that are individual's desires for services on the basis of their own understanding. Some of the perceived needs turn into demands and are titled as expressed needs (4).

Sexual and reproductive health (SRH) rights emphasize on the equality of men and women in the possession of healthcare (5). Despite this fact, only women have been considered the target group for the SRH programs (6). Achieving the millennium development goals involves more than encouraging men to take responsible sexual behaviors. It requires a careful understanding of their concerns about the SRH (7). Men need a wide range of SRH care services, especially education and counseling that are influenced by social and demographic factors. However, healthcare policies for men have mostly focused on their role in women's health, that is, social concerns about sexually transmitted diseases (STD) and unwanted pregnancies. In this respect, less attention is paid to their own SRH care needs. The most important reason for such a negligence is inadequate information about men's needs (8, 9).

SRH services for men consist of clinical services for prevention, screening, diagnosis, and treatment of STDs, sexual dysfunction, infertility, and genital cancers. Indeed, counseling and education regarding skills related to risk assessment, resisting peer pressure, self-concept, decision-making, values, communication, and relationships should be considered (9, 5). Developing proper strategies compatible with the culture and context for meeting men's SRH needs calls for the careful recognition of their actual needs. In the absence of a proper needs assessment, the accomplishment of objectives, resource allocation and the best strategy for addressing these needs are impossible (10). According to reports, education and counseling for men can correct their behavioral patterns including the reduction of domestic violence, high-risk sexual behaviors, drug abuse, STDs, as well as the increase of condom use (11).

In southwestern Asian countries, men encounter plenty of unmet SRH needs (12); however, healthcare providers and researchers often ignore men's health-related needs in these countries (13). For example, in Iran, 56.1% of men suffer from erectile dysfunction (14) and 27.94 per 100 pregnancies are unwanted (15). The men's role in the transmission of STDs and in turn the influence of STDs on men's health are also important. It has been shown that unsafe sexual behaviors are more frequent in men than women (8). It is estimated that 71,000 people are living with Human Immunodeficiency Virus in Iran, of which more than 90% are men (16, 17).

Iran's healthcare system has focused on maternal and child healthcare for decades with men's SRH remaining largely unexplored. There is a remarkable difference in the studies about men's SRH needs and those of women. Available studies on men's SRH needs are limited to their role in family planning at the time when the country's population policy was focused on birth control. Therefore, a little information is available on men's SRH conditions and their related needs (18). The aim of this study was to identify men's SRH needs. The findings of this work can therefore be used for policy-making with the aim of improving men's SRH.

## 2. Materials and Methods

### Study design and setting

The data for the present study came from a research project aimed to assess men's SRH needs and conducted among adult men aged between 20 and 60 years in 2014 in Ahvaz, a major industrial city in the south-west of Iran with many immigrants from different parts of the country. This report is focused on self-reported needs regarding SRH care services. The normative needs (by assessing men's knowledge and attitudes toward SRH) has been published formerly (18).

### Inclusion and exclusion criteria

Men aged between 20 and 60 yr were included in the study and those with unstable mental health were excluded.

### Sample size

Due to the lack of a similar study, a pilot study was conducted on 40 eligible men; their various SRH needs were between 41% and 85%, so for the maximum sample, *p* was considered with the maximum value (0.5) and *d* was considered with the lowest value (0.03).

$$n=\frac{z_{1-\alpha /2}^{2}p\left(1-p\right)}{d^{2}}$$

$$n=\frac{{\left(1.96\right)}^{2}\times 0.5\times 0.5}{{\left(0.03\right)}^{2}}=1068$$

For 95% confidence interval, $z_{1-\alpha /2}=1.96$.

### Data collection and tools

To design the data collection tool, a thorough literature review led to an item pool and the initial questionnaire was developed. The validity and reliability of this questionnaire were assessed using the face validity and content validity. The face validity was performed using both quantitative and qualitative methods. In the qualitative method of face validity, 10 eligible men were requested to assess the tool in terms of difficulty, relevance, and ambiguity of the items, which led to some modifications. Next, the impact score was calculated for each item for modifying or deleting inappropriate items. The items with an impact score of 1.5 or higher were considered suitable and preserved. For content validity, the content validity index and content validity ratio (CVR) were assessed.

For the CVR, 10 faculty members (reproductive health, nursing, and urology) were asked to assess the necessity of each item with a 3-point scale. According to Lawshe table, items with a CVR more than 62% were evaluated as necessary and preserved. For the content validity index, the faculty members commented on relevance, simplicity, and clarity of the items on the basis of Waltz and Bausell Index. Therefore, items with the score of 0.79 or higher were preserved. With regard to reliability, 40 men with the same inclusion criteria included in this study filled out the questionnaire. The internal consistency of the questionnaire was assessed using the calculation of the Cronbach's alpha coefficient, which was reported as 85%. After performing the test-retest method with a 2-week interval, the Pearson's correlation coefficient was reported as 0.89.

The final questionnaire contained questions about self-reported perceived (felt) needs (individual's desires for services on the basis of their own understanding), expressed needs (the felt needs which have turned to demands) (4), and unmet needs (the felt needs which were not fulfilled) (3), and satisfaction with received services. The needs were categorized into clinical services (preventive, diagnostic, therapeutic) and educational/ counseling services. Participants were asked to answer if they had felt the need of any SRH care services within the previous 12 months but did not receive them (3).

For the purpose of data collection, two male interviewers were employed after receiving instructions. A cluster random sampling method was used to recruit the participants. The whole city was divided into five regions (northern, southern, central, western and eastern). Given the population of these regions, some clusters were randomly chosen from each region. Taking into account the proportion of the sample size, 13 blocks of each region and 18 men from each block were chosen. Based on the estimated number of families residing in each block, the number of doorbell buttons was selected. The calculated number was divided into 10 to find the interval number. For instance, if the number of families was 70, the interval number was considered 7. The interviewers referred to the southeastern portion of the block, and the first questionnaire was filled out at the first house. Taking a round of the block clockwise, the interval number (for example, 1+7=8) helped with the selection of the next neighboring house. If a man was not at home, another man would be replaced.

### Ethical consideration

This study was approved by the ethics committee affiliated with Ahvaz University of Medical Sciences (decree number: ajums.REC.1392, 309). The participants were provided with information regarding the aim and process of the study. Also, they were assured about the anonymity and confidentiality of the collected data. Those men who were willing to take part in this study signed the written informed consent form.

### Statistical analysis

Descriptive and inferential statistics were used for data analysis via the SPSS (Statistical Package for the Social Sciences, version 19.0, SPSS Inc., Chicago, Illinois, USA). To investigate the factors influencing the needs for receiving SRH care service, the Chi-squared test and linear regression analysis were used. All variables were entered into a multivariate logistical regression model. The Crude and adjusted odds ratio with a 95% confidence interval was calculated; p< 0.05 was considered statistically significant.

## 3. Results

The mean age of the men was 33.5±9.085 yr. Most of them were married (71.3%), had a high-school education level (42.1%), and an insufficient monthly income (50.7%) (Table I).

The first three priorities of men's perceived, expressed, and unmet needs for SRH clinical services were screening and diagnosis of male genital cancers (63.3%), contraception (36%), and diagnosis and treatment of sexual dysfunction (86.9%), respectively (Table II). Men's SRH educational and counseling needs were preventing STD, HIV/AIDS (72.1%), using contraception correctly (39.5%), and resisting peer pressure (86.6%) as perceived, expressed, and unmet needs, respectively, (Table III).

Associations between the men's needs for receiving SRH care services and demographic factors were shown in Table IV. The men's needs for receiving SRH care services vary in different age groups. Men aged between 51 and 60 years felt the need to receive diagnostic and therapeutic services for STDs and sexual dysfunction more compared with men aged between 20 and 30 years (adjusted odd ratio (AOR) 3.25 [95% confidence interval (CI)=1.72, 6.14], AOR 3.21 [95% CI=1.67, 6.19], respectively). Needs for receiving cancer services in the men aged between 41 and 50 years were lower than the other age group (AOR 0.54 [95% CI=0.34, 0.86]). The married men felt the need for sexual dysfunctions lesser than the unmarried group (AOR 0.37 [95% CI=0.25, 0.55]). Those men who had high school or higher education levels felt fewer needs to receive services for genital cancers and other services. Those men with a higher income felt more needs for receiving services for male genital cancers, STDs/HIV/AIDS, and sexual dysfunctions compared with those men who had no income (AOR 2.64 [95% CI=1.40, 4.29], AOR 2.23 [95% CI=1.27, 3.90], and AOR 1.84 [95% CI=1.06, 3.20], respectively) (Table IV).

Also, 83% of the men did not know where they could ask for SRH care services; 87.5% did not know where they should go if they wanted to have a vasectomy. Moreover, 93% of the men had never received any education regarding SRH care services. More than 95% of the men rated SRH care services as undesirable. Some reasons for such a negative assessment were the absence or inaccessibility (76.1%) and low quality (71.4%) of SRH care services, lack of professional healthcare personnel (55.4%), and reluctance to receive SRH care services (37.8%).

**Table 1:** Socio-demographic characteristics of participants.


**Variable**	**F (%)**
Age	
20–30	507 (47.5)
31–40	350 (32.8)
41–50	140 (13.1)
51–60	71 (6.6)
Sum	1068 (100)
Education	
Elementary	96 (9)
Secondary school	213 (19.9)
High school	450 (42.1)
Associated with art	189 (17.7)
Bachelor of Science	120 (11.2)
Sum	1068 (100)
Marital status	
Married	762 (71.3)
Single	306 (28.7)
Sum	1068 (100)
Premarital sex (single)	
Yes	120 (11.2)
No	186 (17.5)
Sum	306 (28.7)
Sexual partner (single)	
Homosexual	6 (5)
Heterosexual	
Girlfriend	85 (70.8)
Female Sex worker	13 (10.9)
Other	16 (13.3)
Income*	
No income	120 (11.2)
Low	213 (19.2)
Middle	542 (50.7)
High	193 (18.2)
Sum	1068 (100)

Notes: $1 US = 32000 Rials at the time of the study;

Low [< $156 US];

Middle [$156 US–$312 US];

High [> $312 US].

**Table 2:** Needed men's SRH services.


**Priority**	**Perceived (Felt) Needs**		**Expressed Needs (Demands)**		**Unmet Needs**	
	Service	F (%)	Service	F (%)	Service	F (%)
1	Screening and diagnosis of male genital cancers	676 (63.3)	Contraception	384 (36)	Diagnose and treatment of male sexual dysfunction	928 (86.9)
2	Diagnose and treatment of genitourinary diseases *	655 (61.3)	Diagnose and treatment of STDs, HIV/AIDS	364 (32.4)	Diagnose and treatment of genitourinary diseases	917 (85.9)
3	Diagnose and treatment of STDs, HIV/AIDS	589 (55.1)	Diagnose and treatment of genitourinary diseases	318 (29.8)	Screening anf diagnosis of male genital cancers	912 (85.4)
4	Diagnose and treatment of male sexual dysfunction**	581 (54.4)	Diagnose and treatment of male sexual dysfunction	250 (23.4)	Diagnose and treatment of male infertility	901 (84.4)
5	Diagnose and treatment of male infertility	437 (40.9)	Diagnose and treatment of male infertility	245 (22.9)	Diagnose and treatment of STDs, HIV/AIDS	842 (78.8)
6	Contraception	432 (40.5)	Screening and diagnosis of male genital cancers	181 (16.9)	contraception	701 (65.6)

Notes: Descriptive and inferential statistics were used for data analysis;

*Varicocele, hydrocele, prostatic hypertrophy;

**Desire disorder, erectile disorder, orgasm disorder, ejaculation disorder;

STD: Sexual Transmitted Disease; HIV: Human Immune Virus; AIDS: Acquired Immune Deficiency Syndrome.

**Table 3:** SRH-related skills men need (educational & counseling services).


**Priority**	**Perceived (Felt) Needs**		**Expressed Needs (Demands)**		**Unmet Needs**	
	Skills	F (%)	Skills	F (%)	Skills	F (%)
1	Preventing STDs, HIV/AIDS	770 (72.1)	Using contraception correctly	422 (39.5)	Resisting peer pressure	924 (86.6)
2	Resisting peer pressure	622 (58.2)	Preventing STDs, HIV/AIDS	360 (33.7)	Abstinence	918 (86)
3	Fatherhood role	610 (57.1)	Relationship with partner	316 (29.6)	Fatherhood role	909 (85.2)
4	Using contraception correctly	571 (53.5)	Resisting peer pressure	267 (25)	Preventing STDs, HIV/AIDS	840 (78.7)
5	Relationship with partner	554 (51.9)	Fatherhood role	250 (23.4)	Relationship with partner	780 (73.1)
6	Abstinence	527 (49.3)	Abstinence	170 (15.9)	Using contraception correctly	696 (65.2)

Notes: STD: Sexual Transmitted Disease; HIV: Human Immune Virus; AIDS: Acquired Immune Deficiency Syndrome;

AOR: Adjusted Odds Ratio.

**Table 4:** A factor associated with men's perceived (felt) needs for receiving SRH services.


**SRH Service**	**Screening & diagnosis of male genital cancers**		**Diagnose & treatment of STDs, HIV/AIDS**		**Diagnose & treatment of Male sexual dysfunction**	
**Variables**	**Yes**	**AOR (95% CI)**	**Yes**	**AOR (95% CI)**	**Yes**	**AOR (95% CI)**
Age						
20–30	332 (65.5)	1.00	278 (54.8)	1.00	309 (60.9)	1.00
31–40	213 (60.9)	0.80 [0.57, 1.14]	188 (53.7)	1.54 [1.09, 2.16]	149 (42.6)	0.70 [0.50, 0.98] ${}^{*}$
41–50	77 (55.0)	0.54 [0.34, 0.86]${}^{*}$	69 (49.3)	1.13 [0.72, 1.76]	67 (47.9)	0.87 [0.56, 1.34]
51–60	54 (76.1)	1.16 [0.61, 2.23]	54 (76.1)	3.25 [1.72, 6.14]${}^{***}$	56 (78.9)	3.21 [1.67, 6.19]${}^{**}$
Marital status						
Single	202 (66.0)	1.00	203 (66.3)	1.00	210 (68.6)	1.00
Married	474 (62.2)	0.76 [0.51, 1.23]	386 (50.7)	0.30 [0.20, 0.45]	371 (48.7)	0.37 [0.25, 0.55]${}^{***}$
Educational status						
Elementary	77 (80.2)	1.00	51 (53.1)	1.00	55 (57.3)	1.00
Secondary school	159 (74.6)	0.62 [0.34, 1.13]	145 (68.1)	1.53 [0.91, 2.58]	140 (65.7)	0.99 [0.58, 1.67]
High school	262 (58.2)	0.29 [0.17, 0.51]${}^{***}$	237 (52.7)	0.85 [0.53, 1.35]	228 (50.7)	0.63 [0.39, 1.02]
Associated with art	113 (59.8)	0.29 [0.16, 0.54]${}^{***}$	102 (54.0)	0.83 [0.49, 1.40]	103 (54.5)	0.76 [0.44, 1.30]
Bachelor of Science	65 (54.2)	0.23 [0.12, 0.45]${}^{***}$	54 (45.0)	0.55 [0. 31, 0.98]${}^{*}$	55 (45.8)	0.55 [0.31, 0.99]${}^{*}$
Income						
Without income	64 (53.3)	1.00	67 (55.8)	1.00	65 (54.2)	1.00
Low	152 (71.4)	2.12 [1.29, 3.48] ${}^{**}$	122 (57.3)	1.31 [0.80, 2.15]	141 (66.2)	2.45 [1.48, 4.05]${}^{***}$
Middle	334 (61.4)	1.50 [0.92, 2.45]	285 (52.6)	1.42 [0.86, 2.32]	279 (51.5)	1.79 [1.09, 2.94]${}^{*}$
High	126 (65.3)	2.64 [1.40, 4.29] ${}^{**}$	115 (59.6)	2.23 [1.27, 3.90] ${}^{**}$	96 (49.7)	1.84 [1.06, 3.20]${}^{*}$

Notes: Data presented as *n* (%);

*Statistically significant at *p* < 0.05; **Statistically significant at *p* < 0.01; ***Statistically significant at *p* < 0.001;

STD: Sexual pansmitted disease; HIV: Human immune virus; AIDS: Acquired immune deficiency syndrome.

## 4. Discussion

The results of this study showed that the study population had a wide range of unmet needs for receiving SRH care services. This bodes an apparent inequality in access to SRH care services across the world. For instance, in England, free healthcare services are delivered to patients for HIV/STDs, sexual dysfunction, sexual abuse, and vasectomy without the need for referral (19). On the other hand, in the USA, less than half of men received SRH care services in the past year, and the level of the unmet needs for such services among those men who had highly unsafe sexual behaviors was high (32-63%) (13). Also, just a few men had received birth control counseling/methods, including condoms (12%) and STD/HIV testing/STD treatment (12%) (20). In African countries also, the rate of men's access to HIV-related health care services is very low (1–9%) (21). Similar to our findings, a study in the United States showed that the top three perceived needs of men were STD risk, testicular cancer, and HPV vaccine, respectively (22).

In this study, there was no accordance between the priorities of the perceived and expressed needs reported by men. For example, although the first priority of the men's perceived needs was genital cancers, the first expressed need was receiving contraceptives, and demands for services related to the diagnosis and prevention of genital cancers were among the last priorities of expressed needs. This reflects the health policy and accessibility or inaccessibility of services: lack of a framework for offering SRH services or referral system for men, and in turn high accessibility of free family planning services due to country's population policy (until 2013 focused on birth reduction) in Iran's primary healthcare system; since men have no position in Iran's health care system, men's contraceptive methods are also offered to their wives; and single men are therefore neglected.

Cancer is the third top cause of death in Iran with an incidence rate of 110.43 per 100000 among men in 2010 (23). The annual cancer incidence rate in men has increased by 53.0% (24). This highlights the need for the provision of education and clinical care. The assessment of knowledge and attitudes of Iranian men regarding SRH healthcare services showed a lack of knowledge in all aspects including male genital cancers (18). This report identified that the screening and diagnosis of male genital cancers were the first perceived priorities by the men. Nevertheless, according to the WHO's guidelines for cancer screening and prevention, a few national programs are available in Iran (23). In developed countries, cancer-screening programs were increased during the recent decade. The prostate cancer screening was 56% in the USA and 50% in Canada (25, 26).

The diagnosis and treatment of STD/HIV/AIDS have also been among the priorities of men's expressed needs. Although the number of new cases of HIV has begun to decrease, sexual transmission is increasing, for instance, from 1986 to 2011, the number of HIV incidents caused by sexual transmission increased from 11 to 21.1%, often referred to as the `third wave' of AIDS (27). Following the national and universal commitment for fighting AIDS, some free but few centers for diagnosis and treatment of AIDS were established in Iran; however, due to the cultural sensitivities, these centers are not working quite openly but under the title of `counseling centers for behavioral diseases'. Most people are unaware of the presence of such centers, and the activities of such centers are almost limited to the provision of healthcare services to HIV-infected people instead of focusing on prevention in uninfected people. Hence, many problems are encountered providing services to PLHIV (28).

It was found that resisting peer pressure and abstinence were other priorities of SRH unmet skills of men, which were the most important sexual education skills. In this study, 11.2% of unmarried men reported that they had sexual relationships, but the real value is probably much higher. This value in university students was 33 percent for males in 2008 and 23 percent for females in 2011 (29, 30). Evidence show that extra-marital sexual relationships are increasing in Iran (31). Like other Muslim countries, provision of sexual health education for unmarried people is socially unacceptable because of religious and cultural prohibitions (31). Therefore, teenagers are deprived of sexual education and they are neither prepared at homes nor at schools on sexual life. Schools also have neither the commitment nor the qualification to offer such education. Related challenges have mostly a socio-cultural nature and are not caused by religious prohibitions (29). Motamedi and colleagues showed that men had more liberal and permissive attitudes toward premarital sexual relationships compared with women. They concluded that inter-cultural communications led to socio-cultural changes in women and men's sexual attitudes (32).

In this study, the first priority of men's unmet needs was sexual dysfunctions. Evidence shows that sexual dysfunctions among men have become much more prevalent than before (33). While public health programs are focused on HIV, studies in South Asia show that men are more worried about their psychosexual conditions including sexual dysfunction rather than AIDS and STDs (7). The results of this study indicated that the weaknesses in the Iranian healthcare system hindered the provision of such services to men.

This study also revealed that 85.2% of men had unmet needs with regard to fatherhood skills. So far, many studies have been conducted on women's attitudes, behaviors, and experiences on their maternal roles. However, very few studies have been carried out on men's role as father and their paternal responsibilities. Fatherhood is a socio-cultural role and can make a significant contribution to men's lives. When men accept their fatherhood role, they also contribute to the gender equality (34). In many countries, there are institutions backed by policy-makers that provide support in educating and encouraging men to fulfill their fatherhood responsibilities and roles (21).

The relationship between perceived needs for receiving SRH care services and socio-demographic characteristics of men reveals the necessity of providing services in accordance with the marital status, age, educational background, and income. Allin and Masseria reported that accessibility depends on the characteristics of clients besides healthcare-related factors (1).

Most of the participants reported dissatisfaction with the SRH care services due to inaccessibility and low quality of services. Another study in Iran revealed that 78% of university students believed that reproductive healthcare services were insufficient. Unawareness, the absence of a proper condition for offering such services, inaccessibility to services led to failure in fulfilling the SRH goals (35). In this study, different socio-demographic factors influence the men's perceived needs for receiving services. But in the `Robert same' study, men's needs did not vary by any participant predisposing or needs factor (22).

This investigation is one of the first–if any – studies on men's needs for SRH care services in Iran conducted with a wide range of age groups in adults. However, there are some limitations in the generalizability of findings to all Iranian men. Indeed, since the items of the questionnaire were extracted from literature and experts' views, the real needs of men might be different. Exploring men's experiences and preferences regarding SHR care services using qualitative methods is suggested.

## 5. Conclusion

The findings of this study inform designing and planning national healthcare programs to improve men's SRH. National standards for men's SRH services should emphasize educational and counseling services based on their actual needs during their lifespan. Effective male-friendly healthcare services with trained qualified male staff need to be formed and standard protocols for preventive health care services should be developed.

##  Conflict of Interest

The authors declare no conflict of interest.
